# Lower-Limb Arthropathies and Walking: The Use of 3D Gait Analysis as a Relevant Tool in Clinical Practice

**DOI:** 10.3390/ijerph19116785

**Published:** 2022-06-01

**Authors:** Alban Fouasson-Chailloux, Pierre Menu, Marc Dauty

**Affiliations:** 1 Service de Médecine Physique et Réadaptation Locomotrice et Respiratoire, CHU Nantes, Nantes Université, 44093 Nantes, France; pierre.menu@chu-nantes.fr (P.M.); marc.dauty@chu-nantes.fr (M.D.); 2 Service de Médecine du Sport, CHU Nantes, Nantes Université, 44093 Nantes, France; 3 Institut Régional de Médecine du Sport (IRMS), 44093 Nantes, France; 4 Inserm, UMR 1229, RMeS, Regenerative Medicine and Skeleton, ONIRIS, Nantes Université, 44042 Nantes, France

Arthropathy refers to the notion of joint diseases. This is a generic term that can be used in case of joint damage, whatever the origin. Arthropathies can have extremely varied causes, as indicated by the international classification of diseases (CIM 9) [1]. Many pathologies are responsible for joint damage, such as osteoarthritis infections, inflammatory rheumatism or blood diseases. From a clinical perspective, joint damage can be evaluated using two different approaches. The first approach is based on the prognosis of the causal disease and its possibilities of cure. If the pathology is life-threatening, arthropathy will occur in the background. Conversely, the second type of approach focuses on the functional and social consequences of this arthropathy. This approach will be favored if the causal pathology is not life-threatening in the short-term, but results in disability. This is particularly relevant in countries with a high standard of living, as the reduction in mortality rate is accompanied by an increase in chronic diseases. This leads to the need to fight the morbidity caused by these pathologies and preserve the patients’ quality of life, for both individual and medico-economic reasons.

In human pathology, the three-dimensional disability assessment model of the International Classification of Functioning, Disability and Health (ICF) allows for an approach concerning the anatomical or organic consequences (the impairment), the performance of individual tasks (the limitation of activity) and the social impact (restriction of participation) [2]. Joint pathologies respond particularly well to this classification due to the disability for which they are responsible [3,4].

Consequently, it could be interesting to functionally apprehend the consequences of these arthropathies when one wishes to evaluate a pathology, with the musculoskeletal in the foreground, especially if this justifies consulting a physician. Indeed, the model centered on histology or imaging, which is essential in the initial phases of pathology research and does not sufficiently consider clinical realities. This is all the more true since the relationship between organ and function is not linear, especially in diseases of the musculoskeletal system [5,6]. Walking is a particularly interesting evaluation criterion due to its preponderant weight in the functional disability reported by patients with musculoskeletal disorders [4]. Indeed, it ranks first among disabling functional complaints, as is the case with osteoarthritis. Its study in clinical research seems to be a good criterion for the evaluation of a pathology or therapeutic management. Thus, many animal studies in mice or rats have focused on the functional consequences of arthropathies for walking [7,8,9]. However, the differences between animal and human walking are numerous, which makes the transposition of the results uncertain and means that clinical studies are necessary in humans.

Human walking is a natural mode of movement, offering humans autonomy in their movements and optimized energy consumption [10]. Walking is a complex function that is both voluntary and automatic and requires several years of learning before maturity is reached. Walking is the result of a repetition of cycles allowing for the forward translation of the whole body [11]. This cycle consists of two distinct phases: a support phase (60% of the cycle) and an oscillating phase (40% of the cycle) [12].

Walking can be assessed according to temporal and spatial criteria. The temporal parameters are provided according to the walking cycle. For example, the stance phase of a limb that corresponds to the heel strike period (approximately 10% of the cycle) is followed by a full stance until heel lift (from 10 to 45% of the cycle) and ends with a period of pre-oscillation with anterior tilting of the concerned limb (from 45 to 60% of the cycle). During the oscillating phase, the concerned limb is not in contact with the ground and performs a posterior–anterior movement to allow for the passage of the step. The alternation of these phases during bipedal walking induces periods of double support where each foot touches the ground: one at the start of the cycle and the other at the end. The spatial parameters are evaluated by distinguishing the right and the left foot but are considered symmetrical in non-pathological individuals. We can, therefore, evaluate the length of the step, its width or its angulation [13]. Most of the time, the non-pathological gait is described as a vertical and horizontal oscillation of the body's center of gravity. A finer analysis makes it possible to evaluate the kinematics (angular variations) of each joint during each stage of the cycle [11].

These phenomena depend on the muscles, which ensure the harmonious control of movements in the space. Their roles are multiple: acceleration, balancing, etc. Thus, muscle activity can be mixed when walking—concentric, eccentric and isometric—ensuring propulsion but also balance and stability [14]. For example, the thigh muscles (quadriceps and hamstrings) play a fundamental role in walking, insofar as the knee alone greatly contributes to the power developed during this activity [15]. The exerted forces significantly increase with speed (from 1.2 W/kg at 4.5 km/h to 5.6 W/kg at 11 km) [15,16]. This means that the knee joint is at high risk of injury [17,18]. These points highlight the need for the integrity and a proper functioning of the musculoskeletal system to ensure “normal” walking. Indeed, we understand that, in theory, an “arthropathy”, due to its painful consequences, joint limitations and amyotrophy, can induce a significant disruption to the walking pattern of a subject.

Three-Dimensional Gait Analysis (3DGA) is generally used in medicine for the objective evaluation of human walking. This can identify possible alterations but also allows for therapeutic choices or can be used to evaluate the effectiveness of a treatment [19,20]. Three-dimensional Gait Analysis is considered the gold-standard instrumental gait assessment [21]. Three-dimensional Gait Analysis is preceded by a clinical assessment focusing on neuro-orthopedic abnormalities. This collection can be used as a basis for the interpretation of the results that were highlighted by 3DGA. The examination is carried out using kinematics sensors, force platforms and electromyography surface electrodes [22]. To allow for these recordings, reflectors are placed on the surface of the skin next to certain articular structures to allow for the development of a biomechanical model. Electromyographic sensors make it possible to record the contractions of certain muscles or muscle groups during walking. During the examination, the patient is asked to walk at a comfortable and spontaneous speed along a walking corridor, which is approximately 10 meters long. In this walking corridor, force platforms are directly integrated into the ground, making it possible to record accelerations during support. These measurements are coupled with high-frequency video recordings for a detailed analysis of the gait cycle. Generally, a few round trips can obtain all the data that are needed by 3DGA. The data from a 3DGA are then analyzed and compared to subjects who are not symptomatic or free from the studied pathologies. These data are as follows:

- The spatial–temporal parameters (speed, cadence, step length, support and oscillation proportions, etc.), which provide an overall view of a subject's gait or gait cycle.
- Kinematic data (movements captured by optoelectronic system), generally presented in the form of joint displacement curves (e.g., flexion/extension) as a function of time, reflect the three-dimensional measurement in space of the angular variations in the joints. They allow for an analysis of each joint at different periods of the gait cycle.
- Kinetic or dynamic data: recordings of the accelerations measured by the force platforms. These data, in association with the kinematic data, can be used to calculate the force and the powers at the various joints of the lower limbs, according to inverse dynamic methods.
- Electromyographic data assess the involvement of the main muscles of the lower limbs during gait cycles and may provide indications during co-contraction or spasticity phenomena.

Data from 3DGA, clinical examination and imaging make it possible to interpret the causes of impaired gait in a patient [23]. All these elements can help to differentiate the primary walking disorder from its consequences in the form of compensation. It has been demonstrated that 3DGA allows for a more efficient approach in the management of musculoskeletal disorders [24]. However, it is difficult to establish a direct and obvious link between the improvement in data from 3DGA and quality of life. Some studies have shown that the improvement in 3DGA parameters could be accompanied by an improvement in the maximum walking distance. However, there are certain limits to this examination, particularly its cost and its lack of accessibility, as well as the need of teams that are particularly trained in the use, modeling, and interpretation of 3DGA.
